# COVID-19 boosters restore virus-specific immune responses in kidney transplant recipients unresponsive to primary vaccination

**DOI:** 10.1038/s44298-026-00178-5

**Published:** 2026-02-27

**Authors:** Yvette den Hartog, Yannick van Sleen, Lennert Gommers, Luca M. Zaeck, Daryl Geers, A. Lianne Messchendorp, Jan-Stephan F. Sanders, Carla C. Baan, Debbie van Baarle, Rory D. de Vries

**Affiliations:** 1 Erasmus MC Transplant Institute, University Medical Centre Rotterdam, Department of Internal Medicine, Rotterdam, The Netherlands; 2 Department of Medical Microbiology and Infection Prevention, University Medical Center Groningen, Groningen, The Netherlands; 3 Department of Viroscience, Erasmus MC, University Medical Center Rotterdam, Rotterdam, The Netherlands; 4 Department of Internal Medicine, Division of Nephrology, University of Groningen, University Medical Center Groningen, Groningen, the Netherlands; 5 Center for Infectious Disease Control, National Institute for Public Health and the Environment, Bilthoven, The Netherlands; 6 Leiden University Medical Center, Leiden, The Netherlands; 7 University Medical Center Utrecht, Utrecht, The Netherlands; 8 Maastricht University Medical Center, Maastricht, The Netherlands; 9 Radboud University Medical Center, Nijmegen, The Netherlands; 10 Amsterdam University Medical Centers, Amsterdam, The Netherlands; 11 University Medical Center Groningen, Groningen, The Netherlands; 12 Department of Laboratory Medicine, Laboratory of Medical Immunology, Radboud University Medical Center, Nijmegen, The Netherlands

**Keywords:** Diseases, Immunology

## Abstract

Kidney transplant recipients (KTRs) often exhibit impaired immune responses to vaccination, necessitating multiple doses to obtain sufficient protection from severe disease. This study compared immunological mechanisms underlying the vaccine-induced response between KTRs who responded to primary vaccination (primary responders) and those who only responded to a booster vaccination (booster responders). Humoral immune responses, including binding and Fc-mediated functionalities, and T cell responses, were generally comparable in primary and booster responders. More in-depth analyses revealed that booster responders had an expanded memory B cell pool and stronger omicron BA.1 neutralization, while primary responders had more IL-21-producing T cells and a distinct SARS-CoV-2-specific CD4 T cell phenotype. Principal component analysis demonstrated that booster responders exhibited a more refined immune network integration. These results suggest that the delayed immune response of booster responders is not functionally impaired and that repeated vaccination is an effective strategy to achieve adequate protection in this population.

## Introduction

Due to the lifelong immunosuppressive therapies required to prevent transplant rejection, KTRs exhibit impaired immune responses to vaccination against pathogens such as hepatitis B, influenza virus, and *Streptococcus pneumoniae*^1^. The implications of this impaired vaccine responsiveness became particularly evident during the COVID-19 pandemic, with studies revealing lower antibody levels and reduced vaccine efficacy in KTRs following vaccination compared to controls^2–7^. Consequently, KTRs remain at an elevated risk for severe disease and complications upon breakthrough infection.

Due to significant variability in SARS-CoV-2-specific immune responses among KTRs after the two-dose priming vaccination —where only a small proportion developed detectable antibodies (primary responders) and most required additional booster doses to respond—health authorities recommended a third vaccination^2,8^. These variations are influenced by differences in immunosuppressive treatment regimens and the total dosage of immunosuppressive drugs administered to these patients. Nonetheless, they cannot be fully explained by these factors, and the exact immunological mechanisms driving these differences remain unclear. Repeated booster vaccination of initially unresponsive KTRs led to an increase in antibody responders^8^, but it is unclear whether KTRs that require multiple vaccinations have a SARS-CoV-2-specific immune response with comparable quality as in primary responders.

Vaccine efficacy in KTRs is currently low due to both reduced vaccine immunogenicity and the limited capacity of the low antibody levels to target antigenically diverse variants of SARS-CoV-2^9,10^. Neutralizing antibodies (nAbs) are considered an important correlate of protection; however, they are susceptible to immune escape by mutations in the receptor-binding domain of the S protein in emerging variants^11–13^. However, non-neutralizing antibodies binding to more conserved regions of the S protein can still mediate protection through Fc-mediated functionalities, such as antibody-dependent cellular cytotoxicity (ADCC) and antibody-dependent cellular phagocytosis (ADCP). These functionalities remain poorly characterized in KTRs^14–16^. Similarly, virus-specific T cell responses target conserved viral epitopes and retain reactivity with different variants, offering broad and durable protection^17,18^. Notably, it has been observed that KTRs who fail to develop detectable antibodies post-vaccination can still mount a SARS-CoV-2-specific T cell response, suggesting a potential compensatory role for cellular immunity^19,20^. However, among KTRs, it remains unknown whether primary and booster COVID-19 vaccination responders differ in their ability to generate virus-specific T cell responses or antibodies functional through Fc functions.

Clarifying the immunological differences between primary and booster responders among KTRs can provide valuable insights into whether administering additional vaccine doses is sufficient for initial non-responders, or if fundamental differences in immune mechanisms necessitate alternative vaccination strategies. In the RECOVAC trial, aimed at studying the immunogenicity of COVID-19 vaccination, 57% of the KTRs showed humoral responses to primary vaccination^2^. Subsequently, subgroups of non-responding KTR did seroconvert following a 3rd dose or 4th dose^8,21^, although more than 25% of KTRs may lack humoral immunity after 4 doses^22^. Here, we compared the immune responses of primary and booster (3rd and 4th dose) responder KTRs by evaluating the magnitude and quality of their virus-specific antibody and memory T cell responses.

## Results

### Cohort characteristics

A total of 80 patients were enrolled for in-depth analyses, with an equal distribution between primary responders (*n* = 40) and booster responders (*n* = 40) (Supplementary Fig. 1). A comparative evaluation of baseline characteristics, including lymphocyte counts, transplant characteristics, and immunosuppressive treatment, revealed no statistically significant differences between the groups (Table 1). As per study design, the number of vaccine doses differed significantly, with booster responders having received three or four COVID-19 vaccines and primary responders two doses (*p* < 0.0001).

**Table 1:** Baseline characteristics

Characteristic	Primary responders (*n* = 40)	Booster responders (*n* = 40)	*p*-value
Sex, no. (%)			1.0^2^
Male	23 (58)	23 (58)	
Female	17 (42)	17 (42)	
Ethnicity, no. (%)			0.06^2^
Caucasian	36 (90)	37 (93)	
Black	3 (8)	0 (0)	
Asian	0 (0)	3 (8)	
Age at time of first dose, (IQR) – yr	58.5 (51.5 to 69.0)	57.0 (50.0 to 67.3)	0.48^1^
BMI, (IQR) - kg/m^2^	26.1 (24.5 to 29.7)	25.8 (22.5 to 28.6)	0.16^1^
Comorbidities, no. (%)			
Diabetes Mellitus	8 (20)	11 (28)	0.60^2^
Coronary artery disease	6 (15)	5 (13)	
Heart failure	0 (0)	2 (5)	0.49^2^
Chronic lung disease	0 (0)	2 (5)	0.49^2^
Malignancy^+^	5 (13)	5 (13)	
Auto-immune disease	5 (13)	3 (8)	0.71^2^
CMV seropositivity, no. (pos/neg/NA)	23/14/3	21/13/6	1.0^2^
eGFR (IQR)- mL/min/1.73m^2^	57.0 (44.9 to 67.8)	53.1 (41.5 to 70.7)	0.76^1^
Serum creatinine (IQR) - μmol/L	114.0 (93.0 to 138.3)	108.0 (95.0 to 142.8)	0.78^1^
Lymphocyte count, (IQR) - 10^9^/L	1.5 (1.2 to 2.2)	1.5 (1.1 to 2.1)	0.49^1^
Primary renal diagnosis, no. (%)			0.28^2^
Primary glomerulonephritis	5 (13)	1 (3)	
Familial/hereditary renal diseases	4 (10)	3 (8)	
Congenital diseases	0 (0)	3 (8)	
Vascular diseases	3 (8)	3 (8)	
Secondary glomerular/systemic disease	2 (5)	1 (3)	
Diabetic kidney disease	0 (0)	2 (5)	
Other	2 (5)	2 (5)	
Transplant characteristics			
First kidney transplant, no. (%)	33 (83)	34 (85)	0.35^2^
Time after last transplantation, (IQR) – yr	6.0 (3.0 to 9.5)	5.0 (2.0 to 9.0)	0.25^1^
Last transplant			
Living, no. (%)	25 (63)	30 (75)	0.33^1^
Immunosuppressive treatment, no. (%)			
Steroids	31 (78)	32 (80)	1.0^2^
Azathioprine	1 (3)	1 (3)	1.0^2^
Mycophenolate mofetil	26 (65)	32 (80)	0.21^2^
Calcineurin inhibitor	35 (88)	34 (85)	1.0^2^
mTOR inhibitor	5 (13)	1 (3)	0.43^2^
Immunosuppressive treatment, daily dose (mg)^#^			
Steroids	5 (5 to 5)	5 (5 to 6.5)	0.83^1^
Mycophenolate mofetil	1000 (125 to 1000)	1000 (500 to 1000)	0.58^1^
Number of immunosuppression			0.52^1^
1	2 (5)	0 (0)	
2	18 (45)	19 (48)	
3	20 (50)	21 (53)	
Number of previous SARS-CoV-2 vaccination, no. (%)			<0.0001^1^
0	40 (100)	-	
2	-	34 (85)	
3	-	6 (15)	

Values are numbers (percentages) for categorical variables and medians (interquartile ranges) for continuous variables.

^1^*p*-value based on the non-parametric test (Kruskal-Wallis) test.

^2^*p*-value based on Fisher’s exact test.

^+^Including melanomas, excluding all other skin malignancies.

#Data available for 28 primary responders and 40 booster responders.

BMI, body mass index; mo, month; yr, year.

### COVID-19 vaccination induces comparable immune responses in primary and booster responders

**Fig. 1:**
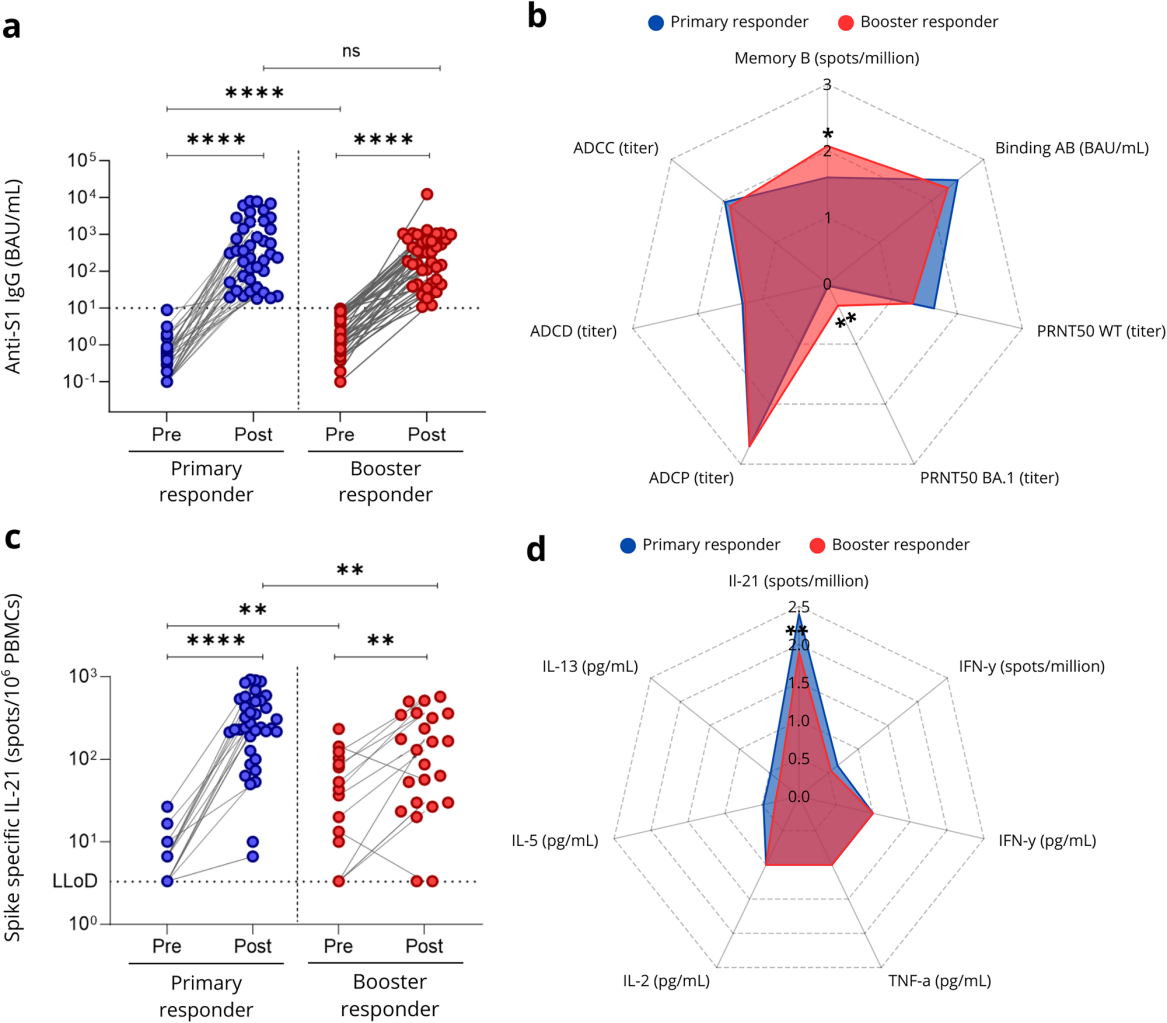
Humoral and Cellular Immune Responses to SARS-CoV-2 Vaccination in primary and booster responders. **a** SARS-CoV-2 S1-specific spike IgG antibody levels at baseline (before priming or booster vaccination) and 28 days after vaccination in primary (blue) and booster (red) responders. The cutoff value for response was 10 binding antibody units (BAU) per milliliter, indicated by the dotted horizontal line. **b** Spider plot illustrating SARS-CoV-2-specific humoral immune responses 28 days after vaccination between primary (blue) and booster (red) responders. **c** SARS-CoV-2 S-specific T-cell IL-21 production at baseline (before priming or booster vaccination) and 28 days after vaccination in primary (blue) and booster (red) responders. The lower limit of detection (LLoD) was 3.3 spots per 10⁶ peripheral blood mononuclear cells (PBMCs), indicated by the dotted horizontal line. **d** Spider plot depicting SARS-CoV-2-specific cellular cytokine production 28 days after vaccination between primary (blue) and booster (red) responders. Spider plots visualize overall immune response profiles. Values represent group means of log10(x + 1)-transformed assay readouts, with radial axes corresponding directly to the log-transformed scale. *P*-values for comparisons between primary and booster responders were calculated using Mann-Whitney tests. Statistical significance is indicated by asterisks: *p < 0.05, **p < 0.01, ***p < 0.001, ****p < 0.0001.

The immunogenicity of COVID-19 vaccination was assessed at 28 days post-vaccination in both primary and booster responders. S1-specific IgG binding antibodies targeting ancestral SARS-CoV-2 increased significantly in both groups (*p* < 0.001), with no significant difference between primary and booster responders at day 28 (Fig. 1a, Supplementary Fig. 2). No significant differences were observed in antibody functionalities, including ancestral neutralizing antibodies, ADCP, ADCC, and ADCD at 28 days post-vaccination (Fig. 1b, Supplementary Fig. 2), with the BA.1 difference driven by a subset of responders in the booster group. Both groups showed a significant increase in SARS-CoV-2-specific IL-21 T cell responses by 28 days post-vaccination, with a significant difference observed between the groups at both pre-vaccination and 28 days post-vaccination (Fig. 1c, Supplementary Fig. 3). Booster responders exhibited significantly higher IL-21 responses at baseline compared to primary responders; however, by 28 days post-vaccination, primary responders had higher IL-21 responses than booster responders. To assess whether pre-existing cellular immunity influenced post-vaccination immune responses, we stratified post-vaccination immune parameters according to each individual’s baseline IL-21 responses (Supplementary Fig. 4). This analysis revealed no dominant effect of pre-vaccination IL-21 levels on post-vaccination immune parameters in either primary and booster responders. No significant differences were observed in concentrations of other cytokines in supernatants of SARS-CoV-2-stimulated cell cultures between the two groups 28 days post-vaccination (Fig. 1D, Supplementary Fig. 3).

### SARS-CoV-2-specific CD4 T cells cluster into three phenotypes

**Fig. 2:**
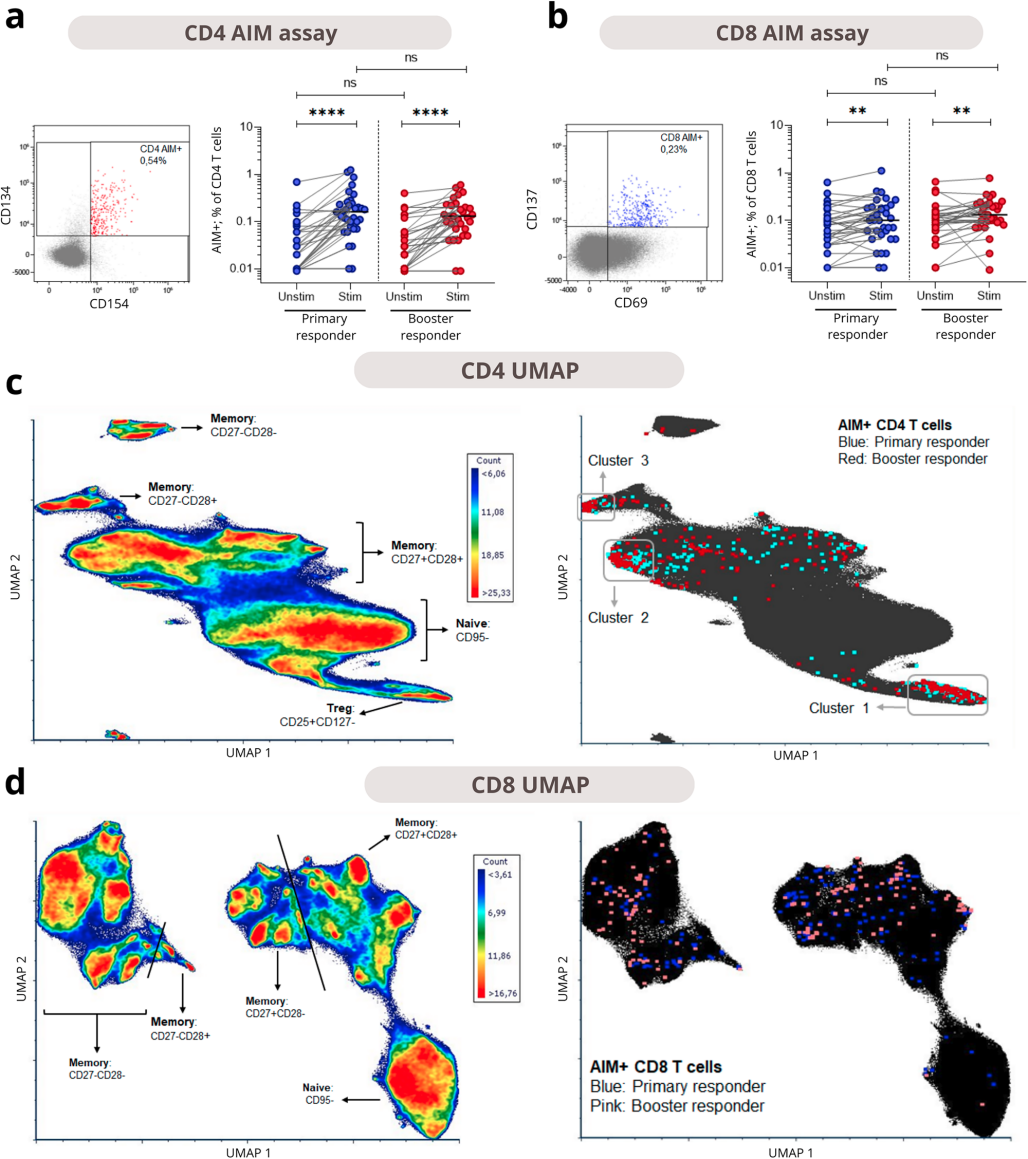
Identification of SARS-CoV-2-specific CD4 and CD8 T cells. Expression of AIM markers was compared in unstimulated and Spike-stimulated samples of primary responders and booster responders. **a** The left panel displays co-expression of CD134 and CD154 by CD4 cells in a stimulated example, indicating AIM+ cells. The right panel shows the induction of AIM+ cells by Spike stimulation in both groups. **b** The left panel displays co-expression of CD69 and CD137 by CD8 cells in a stimulated example, indicating AIM+ cells. The right panel shows the induction of AIM+ cells by Spike stimulation. Medians of the stimulated samples, results of the Wilcoxon signed-rank test between unstimulated and Spike-stimulated samples, and the Mann-Whitney U test between primary and booster responders, are shown in the plots. **c** and **d** display UMAPs of CD4 and CD8 T cells to identify the phenotype of the SARS-CoV-2-specific T cells, in whose generation the AIM markers were excluded. In the left panels, a density plot is displayed with the main subsets indicated. The right plots display the localization of AIM+ cells on the UMAP, indicated by the colored dots. Clusters of AIM+ cells were identified in the CD4 UMAP: Cluster 1 as Tregs, Cluster 2 as CD27 + CD28+ memory cells, and Cluster 3 as a small CD27-CD28+ memory cluster. Statistical significance is indicated by asterisks: *p < 0.05, **p < 0.01, ***p < 0.001, ****p < 0.0001.

Next, an in-depth phenotypic analysis of the CD4 and CD8 T cell response was performed using spectral flow cytometry. SARS-CoV-2-specific CD4 T cells were identified by the co-expression of CD134 (OX40) and CD154 (CD40L, Fig. 2a). Comparing unstimulated and S-stimulated samples, a clear presence of SARS-CoV-2-specific CD4 T cells was observed in both primary and booster responders. Frequencies of these AIM+ cells did not differ between primary and booster responders. AIM + CD8 T cells were also identified (Fig. 2b), based on CD69 and CD137 co-expression. S-stimulated samples had significantly more AIM+ cells than unstimulated samples, though the increase was less pronounced than in CD4 T cells. Similarly, no differences in AIM + CD8 T cells were observed between groups.

To gain more insight into the phenotype of SARS-CoV-2-specific CD4 and CD8 T cells, UMAPs were generated for the CD4 (Fig. 2c) and the CD8 (Fig. 2d) population. Notably, 74% of AIM + CD4 cells in the stimulated samples clustered into three distinct phenotypic groups. The cluster with the most AIM+ cells localized around the CD25^hi^CD127^-^ Tregs (cluster 1), the second-largest cluster of CD4 AIM+ cells comprised CD27^+^CD28^+^ memory cells (cluster 2), and the smallest cluster consisted of CD27^-^CD28^+^ memory cells (cluster 3). CD8 AIM+ cells did not form distinct clusters, and AIM + T cells were evenly distributed across the memory population in the UMAP. This distribution was similar in unstimulated samples (Supplementary Fig. 5**)**.

**Fig. 3:**
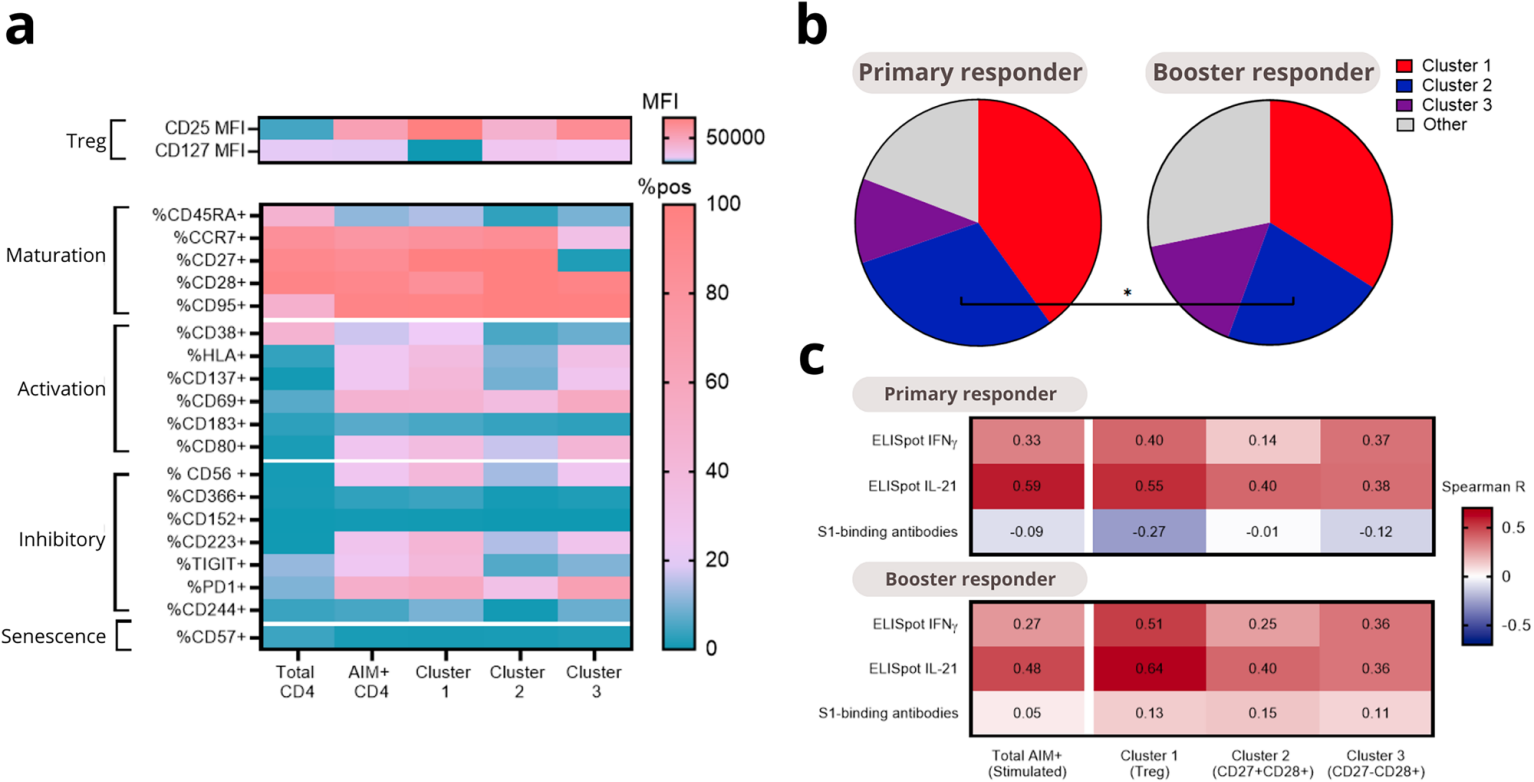
Phenotyping of AIM + CD4 clusters and their correlations with immunogenicity outcomes in primary and booster responders. This figure presents an analysis of the three AIM+ clusters identified in the CD4 UMAP. **a** A heatmap displays the expression of spectral flow cytometry panel markers on total CD4 T cells, total AIM+ cells, and the three specific clusters in stimulated samples. CD25 and CD127 expressions are shown as MFI due to their data distribution, while other markers are presented as percentage positive. **b** Pie charts illustrate the proportion of AIM + CD4 T cells, comparing primary and booster responders. **c** Correlation coefficients of the individual frequency of AIM+ cells and cells in the three clusters with the spot-forming counts in the IFNγ and IL-21 ELISpots and antibody titers at 28 days after vaccination. Statistical testing by Mann-Whitney U and Spearman R. Statistical significance is indicated by asterisks: *p < 0.05, **p < 0.01, ***p < 0.001, ****p < 0.0001.

Next, the three clusters of AIM + CD4 T cells were compared phenotypically, and whether their distribution differed between primary and booster responders. Compared to the total CD4 T cell population, AIM+ cells clearly expressed activation and inhibitory markers (Fig. 3a). Cluster 1, identified as Treg-like, had high expression of inhibitory markers TIGIT, PD-1, CD56, and CD244, but also showed the highest expression of activation markers, including HLA-DR and CD38. Cluster 3 was a mix of cells of clusters 1 and 2, while exhibiting a CD27^-^CD28^+^ effector memory phenotype. Proportions-wise, primary responders had a significantly higher proportion (32% vs 24%) of the CD27^+^CD28^+^ memory cluster 2 than booster responders (Fig. 3b). To better understand the functional relevance of these clusters, we next analyzed associations between the frequencies of the three AIM + CD4 clusters and immunogenicity outcomes (Fig. 3c for IFNγ and IL-21 ELISpot counts, and S1-binding antibody titers, other correlations shown in Supplementary Fig. 6). Correlation patterns were comparable for primary and booster responders. Total AIM + CD4 T cells showed a weak correlation with IFNγ ELISpot counts, a moderate to strong correlation with IL-21 ELISpot counts, and no correlation with antibody titres. All clusters correlated with both IFNγ and IL-21 ELISpots. Together, these findings indicate that SARS-CoV-2-specific CD4 T cells in cluster 1 may phenotypically resemble Tregs, but they are not correlated with functional impairment of T cell responses after vaccination.

### Booster responders have more CD8 TEMRA cells

**Fig. 4:**
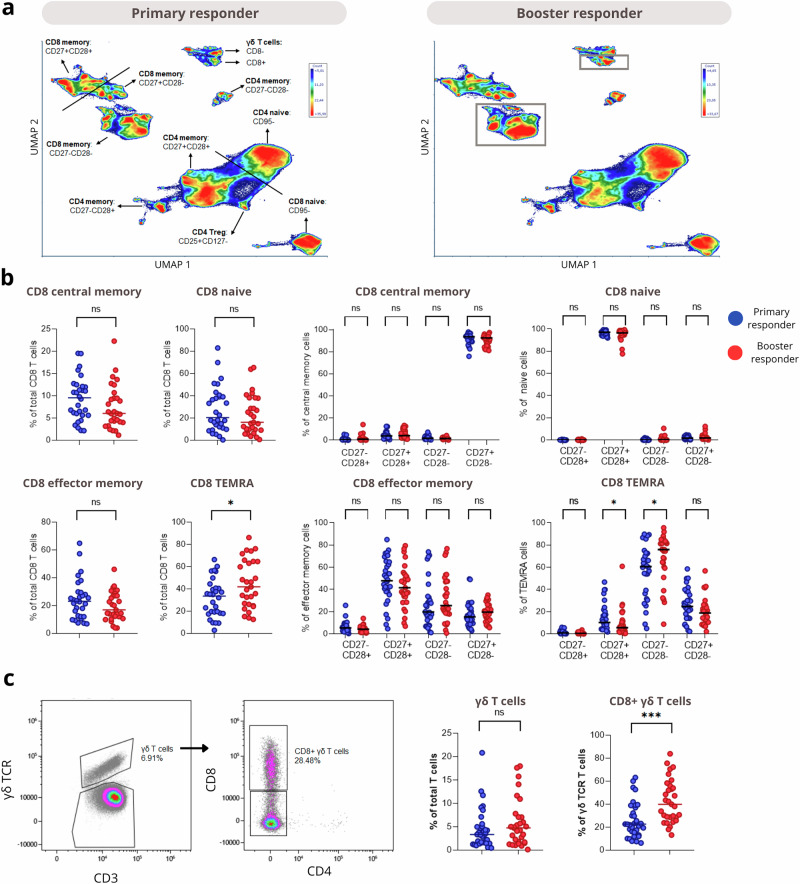
Phenotypical differences in T cell populations between primary and booster responders. Unstimulated samples were compared between primary responders and booster responders. For analyses dependent on CD45RA as a gating marker, six samples were excluded that displayed an aberrant CD45RA expression that is likely associated with C77G polymorphism. **a** Shown are UMAPs of all T cells, with annotated subpopulations in the left plot, and the most deviating populations indicated in the right plot. **b** Comparison of main CD8 T cell subsets between groups. **c** Left panels demonstrate the gating strategy used to identify γδ T cells and their CD8+ subpopulation. The right panels show the quantification of these subsets. Medians and results of statistical testing by Mann-Whitney U are indicated in the graphs. Statistical significance is indicated by asterisks: *p < 0.05, **p < 0.01, ***p < 0.001, ****p < 0.0001.

Next, data from unstimulated samples were analyzed to identify phenotypic differences in T cell phenotypes between primary and booster responders. Several CD4, CD8, and γδ T cell subsets were identified in unsupervised analyses (Fig. 4a), with separation primarily driven by naïve and memory markers (CD45RA, CCR7) and CD27/CD28 expression. Subtle differences in CD8 memory populations and γδ T cells expressing CD8 between primary and booster responders were observed. Manual gating of these subsets revealed that booster responders had significantly more CD8 TEMRA cells (CD45RA^+^CCR7^-^) than primary responders (42% vs 34%; Fig. 4b). Additionally, the groups differed in CD27/CD28 expression within the CD8 TEMRA population, with booster responders exhibiting higher frequencies of CD27^-^CD28^-^ TEMRAs and lower frequencies of CD27^+^CD28^+^ TEMRAs compared to primary responders. Total γδ T cell frequencies were not different between groups (Fig. 4c). Based on the UMAP analysis, we also quantified γδ T cells expressing CD8 and found a significantly higher proportion (*p* < 0.0001) in booster responders than in primary responders. Even though the CMV serostatus has been associated with shifts in CD8 and γδ T cell phenotypes^23–25^, the frequency of CMV seropositive individuals in both groups was exactly similar (62%, Table 1). Additionally, we found that CMV+ individuals had higher frequencies of CD8 TEMRA cells than CMV- individuals, but no differences in CD8-expressing γδ T cells (Supplementary Fig. 7). Finally, CD4 T phenotypes – including Tregs – were found to be similar between groups (Supplementary Fig. 8).

### Stronger correlations between cellular and humoral immune responses in booster responders

**Fig. 5:**
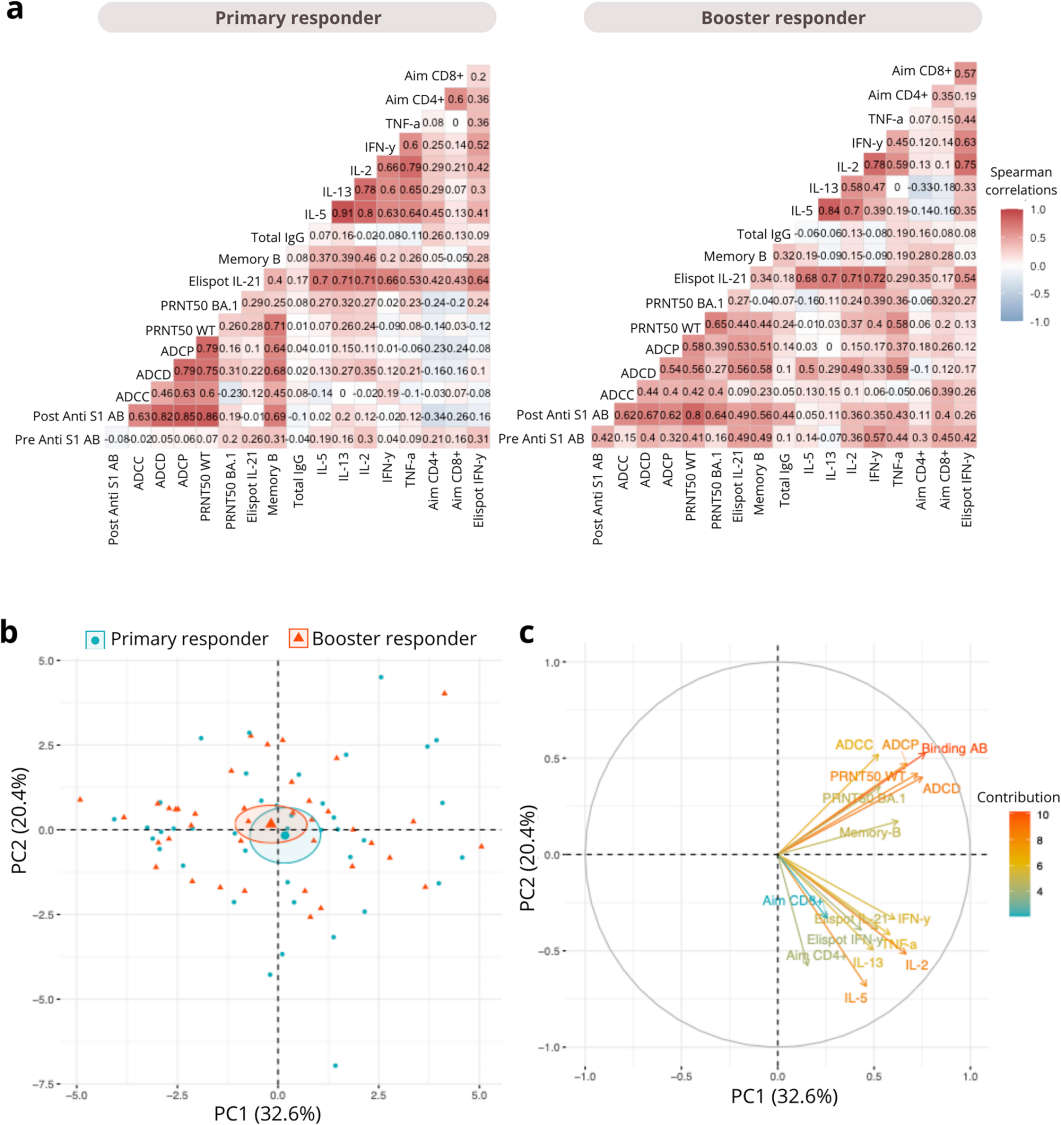
Minor differences in Immune response profiles between primary and booster responders. **a** Correlation matrixes showing Spearman correlations of SARS-CoV-2-specific antibody and T-cell immune parameters 28 days after vaccination for primary and booster responders. Correlations were calculated across all measured parameters, highlighting the relationships between humoral and cellular immune responses. **b** PCA plot of individuals grouped as primary responders (blue circles) and booster responders (red triangles), based on SARS-CoV-2-specific antibody and T-cell parameters. Each point represents a participant, and ellipses indicate 95% confidence intervals for each group. **c** Principal Component Analysis (PCA) of immune variables based on SARS-CoV-2-specific antibody and T-cell parameters. Variables contributing to the PCA dimensions are shown as arrows, with the length and direction reflecting their contribution to PC1 (32.6%) and PC2 (20.4%).

At 28 days post priming or booster vaccination, the coordination of both cellular and humoral immune responses was assessed by investigating the correlation between all immune components (Fig. 5a). In primary responders, moderate positive correlations (r = 0.3 to 0.7) were observed among T-cell activation markers (CD4⁺ AIM, CD8⁺ AIM) and cytokine responses (IFNγ, TNFα, IL‑2, IL‑5, IL‑13), as well as between humoral measures including total IgG levels, memory B-cell frequencies, and functional antibody responses (ADCP, ADCD, ADCC) (Fig. 5b**)**. In booster responders, correlations within the T-cell compartment were higher, and correlations between T-cell responses and humoral responses were increased compared to primary responders. Notably, neutralizing antibody titers and Fc‑mediated functions correlated more strongly with T‑cell cytokine production (Fig. 5a**)**. To better understand the underlying sources of variance contributing to this coordinated network, we performed a principal component analysis (PCA). The first two principal components (PC1 and PC2) accounted for 53% of the total variance, with PC1 explaining 32.6% and PC2 20.4% **(**Fig. 5b**)**. The PCA biplot revealed that both humoral and cellular variables loaded positively on PC1, suggesting this axis reflects a shared trend across immune compartments. However, PC2 separated the two: humoral variables had positive scores on both PC1 and PC2, while cellular variables, despite positive PC1 scores, loaded negatively on PC2. This divergence highlights a distinct dimension of variation between humoral and cellular responses, underscoring their differential contributions to overall immune heterogeneity **(**Fig. 5c**)**.

## Discussion

In this study, we clarified the immunological differences between primary and booster vaccination responders among KTRs, focusing on whether fundamental distinctions exist in the magnitude and quality of virus-specific antibody and memory T cell responses following vaccination. Booster responders were defined as KTRs who seroconverted after a third or fourth vaccination. Our findings reveal that both groups generate comparable immune responses in terms of magnitude by 28 days post-vaccination. Moreover, the quality of SARS-CoV-2-specific immune responses was comparable between primary and booster vaccination responders, although some minor differences could be observed.

To our knowledge, this is one of the first studies to directly compare immune responses between primary and booster responders in KTRs. The overall immune profiles of primary and booster responders were largely similar, indicating that booster vaccination does not generate a fundamentally distinct immune response in these KTR, even though they did not seroconvert upon the first vaccination. To our knowledge, this is one of the first studies to directly compare immune responses between primary and booster responders in KTRs. The overall immune profiles of primary and booster responders were largely similar, indicating that booster vaccination does not generate a fundamentally distinct immune response in these KTR, even though they did not seroconvert upon the first vaccination. The prior study examining humoral and cellular immune responses following primary vaccination in this cohort revealed that some KTRs exhibited discordant responses: 14% were T cell responders without detectable humoral responses, while 28% showed humoral responses without corresponding cellular activation^20^. This discordance was reflected in the weak correlations between humoral and cellular parameters among primary responders. In contrast, booster responders seem to demonstrate stronger associations between these immune compartments. Alternatively, these stronger correlations may simply reflect greater immunological heterogeneity among booster responders, which makes associations between immune parameters more readily detectable. Although the clinical implications of these findings remain to be clarified, our data support the continued administration of booster vaccinations, which enhance humoral and cellular responses to a comparable level.

Despite these similarities, some immune parameters differed between groups. Booster responders exhibited a modest increase in SARS-CoV-2-specific B cells and higher neutralizing activity against the omicron BA.1 variant. However, this expansion in the B cell compartment did not translate into significantly higher levels of binding or neutralizing antibodies, nor was there an enhancement in Fc-mediated effector functions. Although a third mRNA vaccine dose is known to expand the memory B cell repertoire and increase response breadth^26^, the increase in B cell numbers observed here did not improve the functional quality of the humoral response. One possible explanation is the observed reduction in IL-21-producing T cells in booster responders, compared to primary responders. Since IL-21 is essential for B cell differentiation, class switching, and affinity maturation, lower IL-21 levels may underlie the limited antibody output despite expanded B cell numbers^27,28^. Notably, although booster responders had higher baseline levels of IL-21 prior to the booster dose, suggesting residual priming from earlier vaccination, this did not lead to a functional boost in humoral immunity. Together with the weak associations between baseline cellular and post-vaccination humoral parameters within each group, the PCA-supported independence of IL-21 production and memory B-cell responses suggests that pre-existing IL-21 responses do not dictate the magnitude of the immune response following boosting.

This study represents, to our knowledge, the most comprehensive in-depth analysis of T cell responses following COVID-19 vaccination in KTRs. A stronger induction of CD4 than CD8 virus-specific T cells was observed, with similar patterns for both primary and booster responders.

Using spectral flow cytometry, we identified three distinct clusters of virus-specific CD4 T cells, with the largest cluster exhibiting a Treg-like phenotype. This cluster was defined by its CD25^hi^CD127^-^ phenotype, along with elevated levels of markers typically associated with Tregs, such as TIGIT, CD137, and PD-1. Interestingly, this cluster also showed the highest expression of other inhibitory markers (CD56, LAG3, CD244) and chronic activation markers (CD80, CD38, HLA-DR), which could further indicate functional impairment. Recent evidence suggests that not all AIM⁺ CD4 T cells represent true antigen-specific activation; a subset expressing CCR6 and Treg-associated transcription factor FOXP3, may instead represent bystander-activated cells responding to cytokines like IL-2 rather than direct TCR engagement^29,30^. Although CCR6 was not included in our panel, the Treg-like cluster 1 likely overlaps with this CCR6⁺ subset. AIM + CD4 T cells with a Treg phenotype are not exclusive to KTRs; they are also found in healthy individuals^31,32^. However, their frequency appears elevated in KTRs, potentially due to immunosuppressive therapy^33^. Since a Treg phenotype is generally linked to dampened immune responses, including vaccine responsiveness^33,34^, future studies should explore the functional implications of this phenotype and directly compare CD4 AIM⁺ subsets and their CCR6 expression between KTRs and controls. Nonetheless, the frequency of cells in cluster 2 correlated strongly with effector cytokines IFN-γ and IL-21 in the ELISpot assay, suggesting that their presence may still reflect a robust initial vaccine response and potentially signal the activation of other antigen-specific T cells within the microenvironment.

We also observed a higher proportion of SARS-CoV-2-specific CD27^+^CD28^+^ memory T cells among primary responders than booster responders. As most functional vaccine response characteristics were similar in both groups, these minor differences in SARS-CoV-2-specific phenotypes could be interpreted as resulting from differential pathways in both groups that led to similar T cell functioning. Alternatively, it could be that the booster responders’ T cells initially showed a similar response to the primary vaccination but differentiated their phenotype after the booster. However, the frequency of S-specific CD4 T cells with a Treg phenotype was previously shown to be unaffected by the number of vaccine boosts or the timing of blood collection in healthy individuals, suggesting that the observed differences stem from the initial immune response rather than the booster’s additional effect^32^.

In this cohort, age, sex, lymphocyte counts, and immunosuppressive therapy did not differ significantly between primary and booster responders, suggesting that other factors may underlie the observed differential responsiveness to primary vaccination^35^. These could be nutritional status^36–39^, or differences in baseline immune characteristics like inflammation, proliferation, or metabolism^34,40^. To further understand why booster responders failed to respond to the primary vaccination, we performed additional explorative analyses of the total T cell compartment, revealing phenotypic differences in CD8 and γδ T cells between groups. Booster responders had both higher frequencies of CD8 TEMRA cells and more γδ T cells expressing CD8 than primary responders. Additionally, CD8 TEMRA cells in booster responders were more often double negative for CD27 and CD28, consistent with a true TEMRA phenotype^25^. In fact, both these findings suggest more persistent immune activation in the booster responders. This may be caused by microbial stimuli, such as CMV, or damage-associated molecular patterns from the donor organ^23–25^. However, CMV seropositivity did not differ between the two groups, therefore pointing rather to a differential response to persistent stimuli in booster responders.

This study has several strengths, including the use of well-matched cohorts, which ensured comparability between primary responders and booster responders. The extensive analysis of immune parameters provided a detailed understanding of vaccine immunogenicity, utilizing state-of-the-art immunological techniques that enhanced the accuracy and reliability of the findings. Additionally, the novel approach to vaccine immunology, investigating differences between primary and booster responders, offers new insights into vaccine response mechanisms. However, the study faced some limitations, such as the lack of a control group, allowing a better understanding of these patients’ immune response relative to the normal situation. The study suffered from some missing samples, potentially affecting the completeness of the data. Finally, examining the initial response to primary vaccination in booster responders might have provided further context for interpreting the findings.

In conclusion, our study provides significant insights into COVID-19 vaccine-induced immunity in KTRs. Primary and booster vaccination elicited comparable immune responses in magnitude, suggesting that the delayed immune response of booster responders is not functionally impaired, and that repeated vaccination is an effective strategy to achieve adequate protection in this population. Our findings underscore the importance of assessing not only the magnitude of immune responses but also the interdependencies between compartments, which could help optimize future vaccination strategies. This is relevant not only for potential future vaccine campaigns against SARS-CoV-2 but also for new vaccine developments against other pathogens.

## Methods

### Study design

This in-depth immunological analysis was conducted on samples obtained from KTRs selected from two clinical studies within the Dutch Renal Patients COVID-19 VACcination (RECOVAC) study. In the first study, performed between February 1^st^ and May 31st, 2021, KTRs received two priming COVID-19 vaccinations. In the second study, which took place between October 20th, 2021, and February 5th, 2022, KTRs that did not seroconvert after the priming vaccine doses received additional COVID-19 vaccinations. For this study, KTRs that seroconverted after two priming COVID-19 vaccinations (primary responders) were selected and matched by age and sex to KTRs that seroconverted after a third or fourth vaccination (booster responders). Both studies were multicentre, conducted at four university medical hospitals in the Netherlands: Amsterdam UMC, UMC Groningen, Radboud UMC Nijmegen, and Erasmus MC Rotterdam. Approval for both studies was obtained from the Dutch Central Committee on Research Involving Human Subjects and the local ethics committees of the participating centres (Medische Ethische Toetsingscommissie AMC, Medische Ethische Toetsingscommissie Erasmus MC, Radboud University Medical Center Executive Board, Medisch Ethische Toetsingscommissie UMCG). This study was conducted in accordance with the Declaration of Helsinki, and written informed consent was received prior to participation.

### Study participants and COVID-19 vaccination

All participants received two doses of the mRNA-1273 COVID-19 vaccine (Moderna Biotech Spain, S.L.) with a 28-day interval between doses, following the manufacturer’s guidelines. Blood samples were collected 28 days after the second vaccination to evaluate the immune response. For the booster study, individuals who did not seroconvert after receiving their second dose were included. These patients were randomly assigned in a 1:1:1 ratio to receive one of three booster vaccination regimens: a single dose of mRNA-1273 vaccine (100 μg, intramuscular), simultaneous administration of two doses of mRNA-1273 in both upper arms (2 × 100 μg, intramuscular), or a single dose of the Ad26.COV2-S vaccine (Janssen Biologics, Leiden, The Netherlands; 5 × 10^10^ viral particles, intramuscular). Similar to the priming study, blood samples were collected 28 days after the booster vaccination to assess immunogenicity. Throughout both studies, individuals with a history of COVID-19 (defined as confirmed by a positive SARS-CoV-2 test or the presence of nucleocapsid-specific antibodies) were excluded. Detailed inclusion and exclusion criteria can be found in the respective publications^2,8^, although importantly, all participants were clinically stable, at least one year post-transplantation, and free from rejection episodes in the preceding year. Data on the type of immunosuppressant therapy were available for every patient; detailed information on the therapy dose was missing for 12 of the primary responders. For this study, 40 age- and sex-matched KTRs from each study were included (Table 1). Immunoassays were conducted using the available samples, which led to differences in the number of patients included for each assay (Supplementary Fig. 1, Supplementary Table 1).

### SARS-CoV-2 S1-specific IgG antibody response

The SARS-CoV-2 S1-specific IgG antibody response in serum samples was measured at baseline and 28 days post priming or booster vaccination using a validated fluorescent bead-based multiplex immunoassay and expressed as BAU/mL. Patients were classified as seropositive or seronegative based on a threshold of 10 BAU/mL, determined by receiver operator curve analysis^41,42^.

### Plaque reduction neutralization test (PRNT)

Neutralizing antibodies against SARS-CoV-2 were evaluated 28 days post-priming or booster vaccination using a plaque reduction neutralization test (PRNT), as previously described^43,44^. Heat-inactivated serum samples were tested against ancestral SARS-CoV-2 and Omicron (BA.1) variants.

### Antibody-dependent cellular cytotoxicity

Antibody-dependent cell-mediated cytotoxicity (ADCC)-mediating antibodies were measured 28 days post-vaccination using an assay for NK cell degranulation as previously described^43^. Briefly, the NK92.05 cell line, genetically modified to express a high-affinity CD16 Fc receptor through a mutation at 176 V (NK92.05-CD16), was used. NK92.05-CD16 cells were cultured in Alpha-MEM supplemented with NaHCO_3_ (2.2 g/L, pH 7.2), 2-mercaptoethanol (0.0001 M), L-glutamine (200 mM), myo-inositol (0.2 mM), 10% horse serum, 10% fetal bovine serum, folic acid (0.004 mM), sodium pyruvate (1 mM), penicillin (100 IU/mL), streptomycin (100 μg/mL), and cultured cells were additionally supplemented with 100 IU/mL recombinant human IL-2 two times a week.

High-binding 96-well plates (Immunolon) were coated overnight at 4°C with 200 ng/well of trimeric prefusion biotinylated ancestral S protein (D614G, Sino Biological) or PBS (background control). Human heat-inactivated sera were 4-log serially diluted in PBS (1:40 to 1:163,840), and a positive control serum pool was included on each plate. After overnight serum incubation, plates were blocked, washed, and incubated with diluted serum at 37 °C for 2 hours. Next, plates were washed, and 100,000 NK92.05-CD16 cells were added with CD107aV450 (1:100, clone H4A3, BD), Golgistop (0.67 μL/mL, BD), and GolgiPlug (1 μL/mL, BD). Cells were incubated at 37°C for 5 hours, washed, and stained with CD56-PE (1:25, clone B159, BD) and LIVE/DEAD Fixable Aqua Dead Cell (AmCyan, Invitrogen, 1:100) at 4 °C for 30 minutes. Cells were fixed with Cytofix/Cytoperm (BD Biosciences) at 4 °C for 30 minutes. Activation of NK92.05-CD16 cells was measured in the CD56 + LIVE gate as the percentage of CD107a+ cells using a FACSLyric (BD). Percentages were adjusted by subtracting background levels measured on the PBS-coated well for the 1:10 dilution. ADCC induction was calculated from the standard curve and expressed as a 50% endpoint titer. When no ADCC induction was observed, the 50% titer was set at 40.

### Antibody-dependent complement deposition

Antibody-dependent complement deposition (ADCD) was assessed 28 days post priming or booster vaccination on FluoSphere NeutrAvidin-labeled microspheres 1.0 µm beads (red, Invitrogen) coated with baculovirus-generated trimeric prefusion biotinylated ancestral (D614G) S protein (Sino Biological) as previously described^45^. For coating, FluoSphere beads were incubated with biotinylated protein in a 1:1 ratio at 37 °C for 2 hours. Following coating, S-coated FluoSpheres were washed 3 times with 5% BSA to block unbound regions on the bead. Beads were stored at 4 °C for up to 48 hours before use in the ADCD assay. Next, heat-inactivated human sera were added to S-coated FluoSphere beads in a 4-log dilution series starting at a 1:10 dilution and ranging to a 1:163,840 dilution, with the mixture then incubated at 37 °C for 2 hours. Next, a 1:50 dilution of guinea pig complement (Sanbio) in Gelatin Veronal Buffer supplemented with Ca2+ and Mg2+ (GVB++, CliniSciences BV) was added to each well and incubated for 15 minutes at 37 °C. To detect complement deposition, an anti-C3-FITC antibody (Fisher Scientific) at a 1:100 dilution was added and incubated for 20 minutes in the dark at 4 °C. C3 deposition on S-coated FluoSpheres was measured as FITC+ FluoSpheres by flow cytometry on a FACSLyric (BD). Background activation was subtracted from S-coated beads incubated with PBS. A serum pool from 19 healthy donors, as described above, was included in each experiment. ADCD titers relative to this standard were calculated and expressed as a 10% endpoint titer. When no ADCD was observed, the endpoint titer was set at 10.

### Antibody-dependent cellular phagocytosis

ADCP was assessed 28 days post-priming or booster vaccination using the monocyte THP-1 cell line, as previously described^45^. THP-1 cells were cultured in RPMI 1640 with 0.05 mM 2-mercaptoethanol, 10% fetal bovine serum, 100 IU/mL penicillin, and 100 μg/mL streptomycin. For the phagocytosis assay, 1.0 μm FluoSphere NeutrAvidin-labeled beads (Invitrogen) were coated with baculovirus-produced biotinylated ancestral (D614G) prefusion trimeric S protein (Sino Biological) by incubating 5 μL beads with 5 μg protein at 37°C for 2 hours. Beads were washed three times with 5% BSA/PBS, resuspended in 0.01% BSA/PBS, and 1.8 × 10^8 coated beads were added per well in a 96-well plate. Serum was serially diluted 1:40 to 1:163,840 in PBS and incubated with beads at 37°C for 2 hours. Then, 50,000 THP-1 cells were added and incubated overnight at 37°C. Cells were stained with CD32-APC (BD) and LIVE/DEAD Fixable Aqua (Invitrogen) and analyzed by flow cytometry (FACSLyric, BD). Phagocytosis was quantified as gMFI of PE-positive cells within the CD32 + LIVE gate (20,000 cells/sample). Background was determined using beads incubated with PBS instead of serum. A standard curve from pooled serum of 19 healthy donors was used to calculate 50% endpoint ADCP titers; undetectable samples were assigned a titer of 40.

### SARS-CoV-2-specific memory B cell response

The SARS-CoV-2-specific memory B cell response was assessed at baseline and 28 days post priming or booster vaccination, using a commercial B cell ELISpot (U-CyTech biosciences) according to the manufacturer’s instructions^46^. Peripheral blood mononuclear cells (PBMCs) were isolated from heparinized blood via Ficoll Paque and stored at −80°C until analysis. Thawed PBMCs (2,000,000 per well) were polyclonally activated with IL-2 and R848 for 7 days. Subsequently, 100,000 cells for SARS-CoV-2-specific and 10,000 for total IgG-producing cells were transferred to a 96-well plate pre-coated with a capture antibody for IgG and incubated for 20 hours. Detection was performed using biotinylated antibodies for IgG and SARS-CoV-2 S, followed by horseradish peroxidase and 3-Amino-9-ethylcarbazole staining. Spots were counted using an ELISpot reader (Bioreader 6000-V; Bio-Sys). The memory B cell response was quantified as the number of SARS-CoV-2-specific IgG-producing cells (spots) per 10^6^ PBMCs. The lower limit of detection (LLoD) of this response was 10 B cell spots per 10^6^ PBMCs.

### SARS-CoV-2-specific IFN-γ and IL-21 memory T cell responses

SARS-CoV-2-specific IFN-γ and IL-21 memory T cell responses were evaluated similarly at baseline and 28 days post priming or booster vaccination using commercial IFNγ (Mabtech) and IL-21 (U-CyTech biosciences) ELISpot assays, according to the manufacturer’s guidelines^20,46^. Thawed PBMCs (200,000 (IFNγ) or 300,000 (IL-21) per well) were added to a 96-well plate pre-coated with an IFNγ or IL-21 capture antibody. Cells were stimulated with SARS-CoV-2 antigens for 20 (IFNγ) or 44 (IL-21) hours using S1 and S2 overlapping peptide pools (15-mer overlapping by 11, of 0.5 μg/mL for each peptide, JPT Peptide Technologies, PM-WCPV-S-1 & PM-WCPV-S-2). Detection involved biotinylated IFNγ or IL-21 antibodies, followed by streptavidin-horseradish peroxidase incubation. To visualize the spots, filtered TMB substrate was used for the IFNγ ELISpot and 3-Amino-9-ethylcarbazole for the IL-21 ELISpot. Spots were counted with an ELISpot reader (IFNγ: AID ELISpot reader; IL-21: Bioreader 6000-V; Bio-Sys). The IFNγ/IL-21 memory T cell response was expressed as the number of SARS-CoV-2-specific cytokine–producing cells (spots) per 10^6^ PBMCs. For each stimulation, the average of the DMSO negative control was subtracted. The LLoD for was 3.3 IL-21 spots per 10⁶ PBMCs, and 5 IFN-γ spots per 10⁶ PBMCs at baseline and/or 28 days post-vaccination.

### SARS-CoV-2-specific T cell cytokine responses

The human Th cytokine panel kit (LEGENDplex, BioLegend, CA, USA) was utilized to quantify cytokines in JPT antigen-stimulated PBMC culture supernatants from the samples of the IL-21 ELISpot 28 days post-vaccination. This panel included interleukin (IL)-2, IL-4, IL-5, IL-6, IL-9, IL-10, IL-13, IL-17A, IL-17F, IL-22, IFN-γ, and TNF-α^19,47^. Culture supernatants were thawed on ice, centrifuged, and prepared as twofold dilutions. These samples were then incubated with monoclonal capture antibody-coated beads for 2 hours. After washing, the beads were incubated with biotin-labeled detection antibodies for one hour, followed by streptavidin-PE for 30 minutes. Following staining, beads were acquired on a BD FACSCanto™ II with BD FACSDiva™ software (BD). Data acquisition and analysis were conducted using LEGENDplex V8.0 software (BioLegend). Cytokine concentrations were determined by assessing the streptavidin-PE signal against a freshly prepared standard curve, expressed as picograms per milliliter (pg/mL) after subtracting the background signal from the negative control. If subtraction resulted in a negative value, it was adjusted to 0 pg/mL.

### T cell spectral flow cytometry

To obtain an in-depth insight into the phenotype of (SARS-CoV-2-specific) T cells, a spectral flow cytometry panel was employed on PBMC stimulated with overlapping S peptide pools. PBMCs were thawed and resuspended at 4 × 106 cells/mL. In each experimental batch, a healthy reference PBMC sample from the same individual was included to control for batch effects. After one hour of resting, 1 × 106 cells were seeded in polypropylene tubes for stimulation. To each tube, 2 µL of anti-CD40 antibody (Miltenyi Biotec, 130-094-133) was added to limit B cell-mediated CD154 (CD40L) internalization upon binding to CD40. Cells were stimulated with SARS-CoV-2 S peptide pools PM-WCPV-S-1 & PM-WCPV-S-2 (JPT Peptide Technologies) at 0.5 μg/mL or equimolar amounts of DMSO (negative control). Cells were cultured for 20 hours at 37 °C, 5% CO_2_. After stimulation, cells were prepared for staining. The panel was based on a panel used previously in the RECOVAC cohort^48^, optimized for staining index in this experimental setting (Supplementary Table 2). PBMCs were washed with PBS before staining for 30 minutes with Zombie UV dye (Biolegend, CA, USA) to detect dead cells. After washing with 3% FCS in PBS, 10 µL of Brilliant Stain Buffer Plus (BD Biosciences) and 5 µL of True stain monocyte blocker were added to each tube. Antibodies were consecutively added to the cells, without washing steps. First, γδTCR, CXCR3, and CD197 (CCR7) antibodies were added one by one, with ten minutes of incubation between each addition. Afterwards, all antibodies labelled with a BUV, BB, or BV fluorochrome were added individually, but without additional incubation time between each antibody. Finally, the remaining antibodies were added simultaneously. After 30 minutes incubation, cells were washed, fixed in 1% PFA, and measured on the Aurora spectral flow cytometer (Cytek Biosciences).

The phenotype of CD4, CD8, and γδTCR T-cells, as well as SARS-CoV-2-specific T-cells, was analysed by conventional gating (Kaluza V2.1 (Beckman Coulter)) and through Uniform Manifold Approximation and Projection (UMAP) analysis (FCS Express 7 software (De Novo Software)). Conventional gating was applied to identify and quantify commonly known T cell phenotypes and activation-induced marker (AIM)+ cells. AIM + CD4 T cells were defined as CD134 (OX40) and CD154 (CD40L) positive, AIM + CD8 T cells as CD69 and CD137 positive. CD45RA expression was monitored with special care to check for individuals with a C77G polymorphism, preventing normal interpretation of CD45RA expression^49^. In the UMAP analyses, for each unstimulated and stimulated sample, single, LIVE CD3+T cells were identified; these data were merged into a unified file with a unique identifier for each sample. An interval down-sampling method was used to select 10,000 cells for each sample; this cell number was achievable in more than 95% of the samples, with the remaining samples having slightly fewer cells included. The UMAP for total T cells was generated using 18 markers, excluding CD3, markers of the AIM assay (CD134, CD154, CD69, and CD137), and markers with a low (< 1% positive) expression (CD366, CD152, CD223, and CD279). The UMAP was constructed following a scaling procedure to standardize the expression values, with the settings: number of neighbors = 50, minimal distance = 0.1, and number of iterations = 2000. To check whether batch differences were still present after correcting the unmixing between batches based on the bridging healthy samples, UMAPs were compared for each batch, and no obvious differences were identified. Finally, two additional UMAPs were designed specifically for pre-gated CD4 T cells and CD8 T cells. A new unified file was made in which 7500 cells or 5000 cells were selected for each sample (CD4 and CD8 T cells, respectively), which was also reached for 95% of the samples. The same settings as for the total T cell UMAPs were applied; additionally, CD4, CD8, and γδTCR were excluded as generating markers.

### Statistical analysis

First, we described the baseline characteristics of the primary and booster responder groups. Categorical variables were reported as numbers (percentages), and differences between groups were assessed using Fisher’s exact test. Continuous variables were presented as medians (interquartile ranges), with differences evaluated using the Kruskal-Wallis test for the alternative vaccination strategies. Second, the levels of S1-specific binding IgG, neutralizing, ADCC, ADCP, and ADCD antibodies were reported. Group differences were assessed using the Mann-Whitney U test or Pearson Chi-square test, depending on the data type and distribution. Additionally, the Wilcoxon Signed Rank test was used to investigate within-group differences. To visualize overall patterns of immune response quality, group mean values of log₁₀(x + 1)-transformed B cell and T cell measurements were displayed in spider plots. Third, Spearman’s correlation coefficient was calculated to explore relationships between S1-specific binding antibodies, neutralizing, ADCC, ADCP, ADCD antibodies, and T cell responses, including IL-21, IL-5, IL-13, IL-2, IFN-γ, TNF-α, and AIM + CD4 and CD8 cells. Correlation matrices were generated to examine these associations while handling pairwise complete observations. This approach ensured robust handling of missing data while maintaining statistical rigor. Fourth, principal component analysis (PCA) was performed to identify patterns in the antibody and T cell responses and reduce dimensionality. Prior to PCA, missing data were handled using multiple imputation via the ‘mice’ package with predictive mean matching (PMM). Five imputed datasets were generated, and one completed dataset was selected for further analysis. A log transformation (log10(x + 1)) was applied to the imputed data to normalize distributions. PCA was then conducted on the transformed dataset with unit variance scaling (standardization) to ensure equal contributions of all variables. The PCA results, including loadings and scores, were visualized using scree plots, score plots, biplots, and variable contribution plots. Additionally, individuals were visualized with confidence ellipses grouped by responder categories to explore clustering patterns in both antibody and T cell responses. Finally, for the spectral flow cytometry analyses, the two responder groups were compared by Mann-Whitney U tests and stimulated and unstimulated samples with the Wilcoxon signed rank test. Based on the CD4 UMAP, three main clusters of AIM + CD4 T cells were identified, which were quantified for each unstimulated and stimulated sample as a percentage of CD4 T cells. Associations of these clusters with the ELISpots and antibody concentrations were assessed by Spearman’s correlation. For the comparison of the total T cell compartment, our comparison was focused on subsets that were driving the UMAP clustering. These subsets were subsequently manually gated and quantified in unstimulated samples. Statistical analyses were performed using GraphPad Prism software version 9.1.2 and RStudio software version 4.0.5. A *p*-value < 0.05 was considered statistically significant.

## Supplementary information

Supplementary Information

## Data Availability

All data used to support the findings of this study are available from the corresponding author upon reasonable request. The underlying code (RStudio software version 4.0.5) for this study is not publicly available but may be made available to qualified researchers on reasonable request from the corresponding author.

## Code availability

The underlying code (RStudio software version 4.0.5) for this study is not publicly available but may be made available to qualified researchers on reasonable request from the corresponding author.
