# Supplementary material for: COVID-19 boosters restore virus-specific immune responses in kidney transplant recipients unresponsive to primary vaccination
Source: Npj Viruses. 2026 Feb 27;4:14. doi: 10.1038/s44298-026-00178-5 (PMC12948986; doi:10.1038/s44298-026-00178-5)
Supplement: Supplementary file 1 — Supplementary Information [file 44298_2026_178_MOESM1_ESM.pdf]

Supplemental Table 1. Assay-specific sample inclusion overview

Immunological assay	Primary responders (n=40)	Booster responders (n=40)
Humoral responses		
Memory B cell	34	25
Binding S1- antibodies	40	40
PRNT50 ancestral	33	39
PRNT50 omicron BA.1	33	39
ADCC	36	40
ADCD	36	40
ADCP	31	35
Cellular responses		
IL-21	36	24
IFN-y	39	29
TNF-a	36	20
IL-2	36	20
IL-5	36	20
IL-13	36	20
T cell phenotyping	34	31

S1: Spike-1, PRNT: Plaque reduction neutralization test, ADCC: antibody-dependent cellular cytotoxicity, ADCD: antibody-dependent complement deposition, ADCP: antibody-dependent cellular phagocytosis, IL: Interleukin, IFN: Interferon

Supplemental Table 2. Markers included in the spectral flow cytometry panel to analyze T cell phenotypes and SARS-CoV-2 -specific T cells

Marker	Conjugated fluorochrome	Quantity used (µL)	Company	Cat code	Clone
CD56	BUV395	4.5	BD	740299	B159
CD28	BUV496	3.5	BD	741168	CD28.2
CD183 CXCR3	BUV563	5	BD	741406	1C6/CXCR3
CD80	BUV615	2	BD	751209	L307.4
CD8	BUV805	0.6	BD	612889	SK1
CD197 CCR7	BV786	1.5	BD	566758	2-L1-A
CD27	VioBright FITC	1.5	Miltenyi	130-113-634	M-T271
CD3	Sparkblue	1	BioL	344852	SK7
CD95	BB700	1	BD	566542	DX2
γδTCR	PerCPeF710	2.5	ThermoF	46-9959-42	B1.1
CD4	cFluorYG584	0.5	Cytek	R7-20041	SK3
CD45RA	Spark NIR 685	2	BioL	304168	HI100
CD127	APC-R700	3	BD	565185	HIL-7R-M21
HLA-DR	BV570	2	BioL	307638	L243
CD134 Ox40	BV605	2	BD	745217	L106
CD69	BV650	1	BioL	310934	FN50
CD137 41BB	PE	6	Miltenyi	130-119-885	4B4-1
CD154 CD40L	PE-Dazzle594	2	BioL	310840	24-31
CD25 IL-2RA	PE-AF700	2.5	ThermoF	MHCD2524	
CD38	APC-Fire810	1	BioL	303550	HIT2
CD366 TIM3	BUV737	3	BD	748820	7D3
CD152 CTLA4	BV421	5	BioL	369606	BNI3
CD223 LAG3	BV480	2	BD	746609	T47-530
TIGIT	BV711	1	BD	747839	741182
CD279 PD-1	BV750	2	BioL	329966	EHI2.2H7
CD244	PE-Cy5.5	1	ThermoF	35-5838-42	eBioC1.7
CD57	APC	1	BioL	359610	HNK-1
Zombie UV dye		100 µL of 1/1000 dilution in PBS			

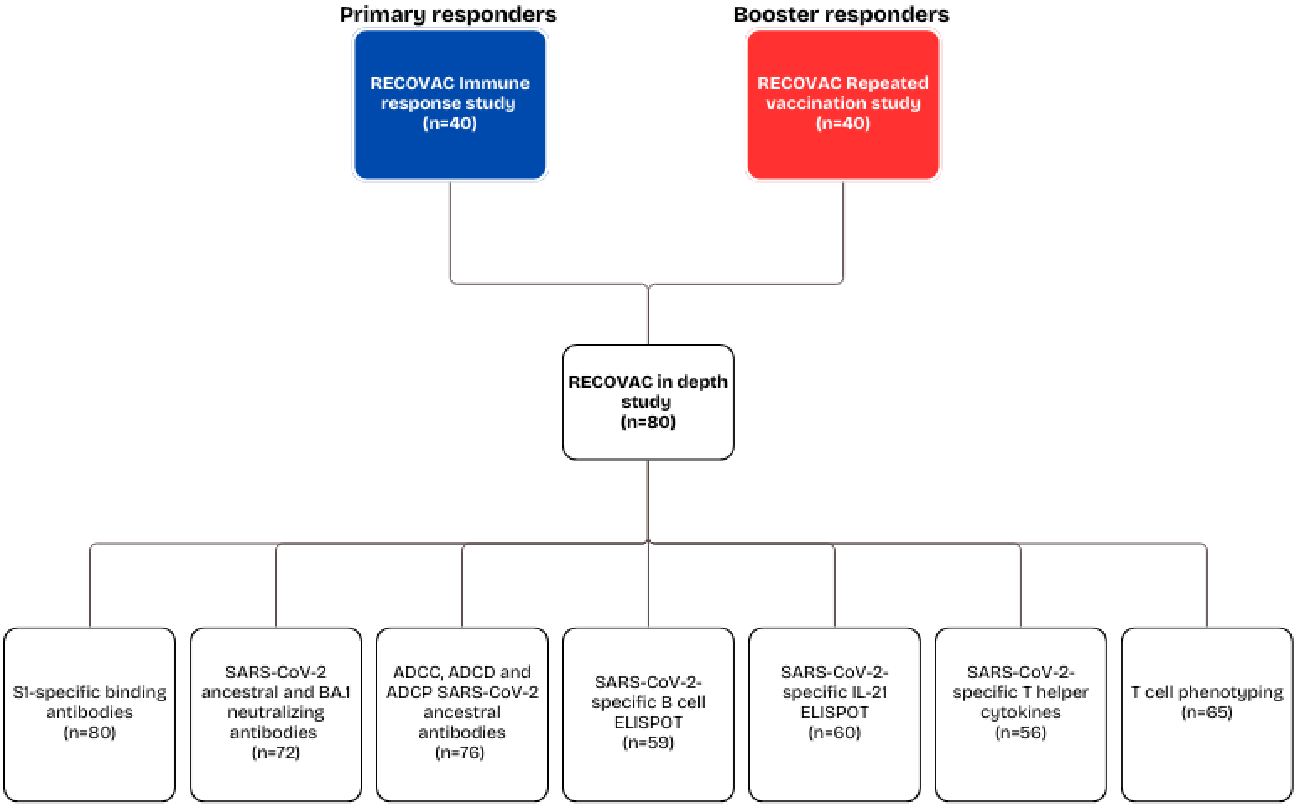

Supplementary Figure 1. Sample selection and inclusion per assay. Overview of subject allocation, and the number of measurements per assay.

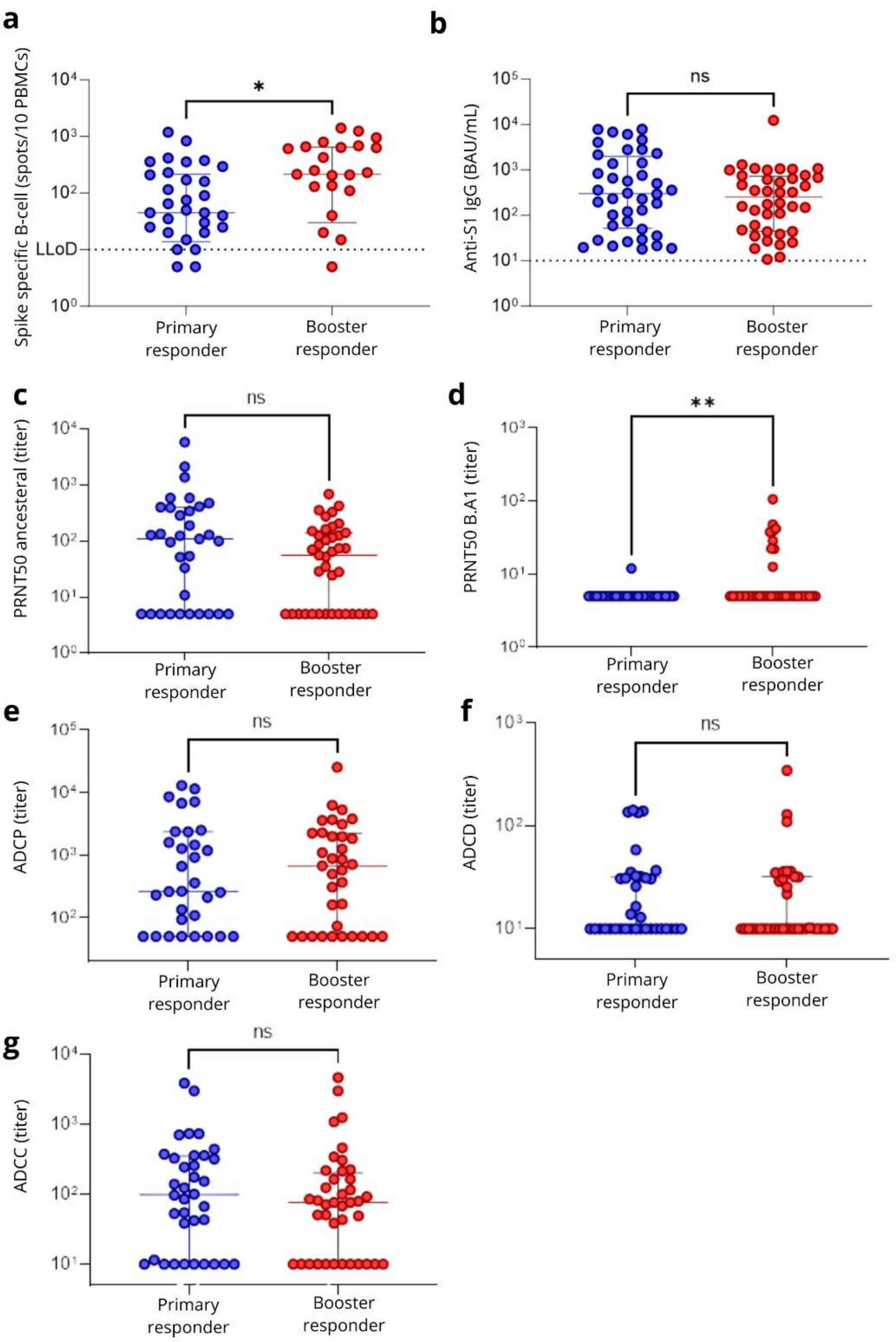

Supplementary Figure 2. Humoral immune Responses to SARS-CoV-2 Vaccination in primary and booster responders. (a-g) Boxplots showing immune responses 28 days post-vaccination in primary (blue) and booster (red) responders. **(a)** SARS- CoV-2 Spike- specific spike B-cells. The lower limit of detection (LLoD) was 3.3 spots per 10^6 peripheral blood mononuclear cells (PBMCs), indicated by the dotted horizontal line. **(b)** SARS-CoV-2 S1-specific spike IgG antibody levels. The cutoff value for response was 10 binding antibody units (BAU) per milliliter, indicated by the dotted horizontal line. **(c)** Neutralizing antibodies against wild-type SARS-CoV-2. **(d)** Neutralizing antibodies against the BA.1 variant. **(e)** Antibody-dependent cellular phagocytosis (ADCP) activity. **(f)** SARS-CoV-2 specific Antibody-dependent cellular cytotoxicity (ADCD) activity. **(g)** SARS-CoV-2 specific Antibody-dependent cellular cytotoxicity (ADCC) activity**.** For PRNT50 assays, zero values were placed at 5 for visualization purposes. P-values for comparisons between primary and booster responders were calculated using Mann-Whitney tests with median and interquartile range shown. Statistical significance is indicated by asterisks: * $p < 0.05$, ** $p < 0.01$, *** $p < 0.001$, **** $p < 0.0001$.

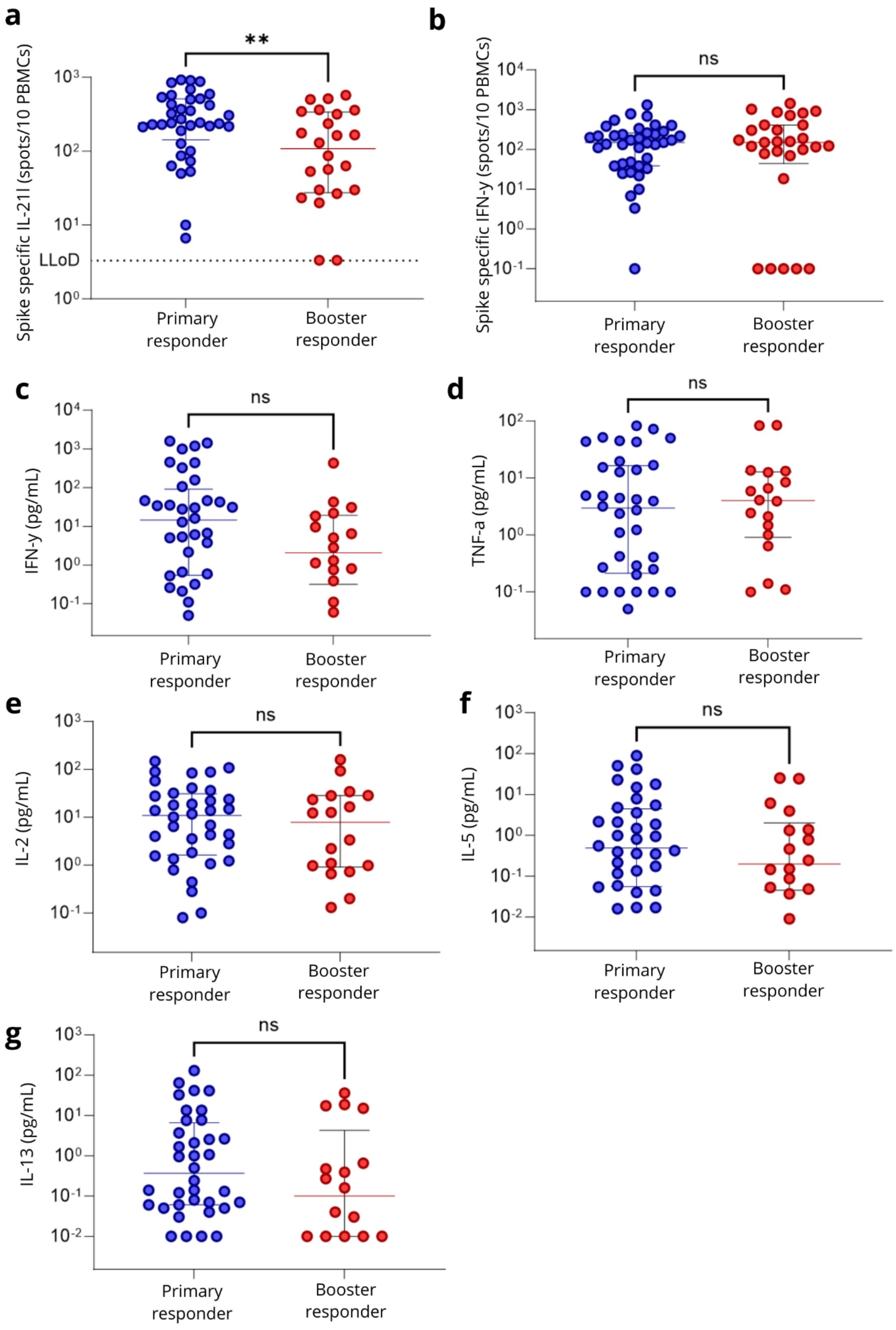

Supplementary Figure 3. Cellular immune Responses to SARS-CoV-2 Vaccination in primary and booster responders. (a-g) Boxplots showing immune responses 28 days post-vaccination in primary (blue) and booster (red) responders. **(a)** SARS- CoV-2-specific IL-21 producing T-cells. The lower limit of detection (LLoD) was 3.3 spots per 10^6 peripheral blood mononuclear cells (PBMCs), indicated by the dotted horizontal line. **(b)** SARS-CoV-2-specific IFN-γ producing T-cells. **(c)** SARS-CoV-2-specific IFN-γ producing T-cells (Legendplex). **(d)** SARS-CoV-2-specific TNF-α producing T-cells. **(e)** SARS-CoV-2-specific IL-2 producing T- cells. **(f)** SARS-CoV-2-specific IL-5 producing T-cells. **(g)** SARS-CoV-2-specific IL-13 producing T-cells. P-values for comparisons between primary and booster responders were calculated using Mann-Whitney tests with median and interquartile range shown. Statistical significance is indicated by asterisks: * $p < 0.05$, ** $p < 0.01$, *** $p < 0.001$, **** $p < 0.0001$.

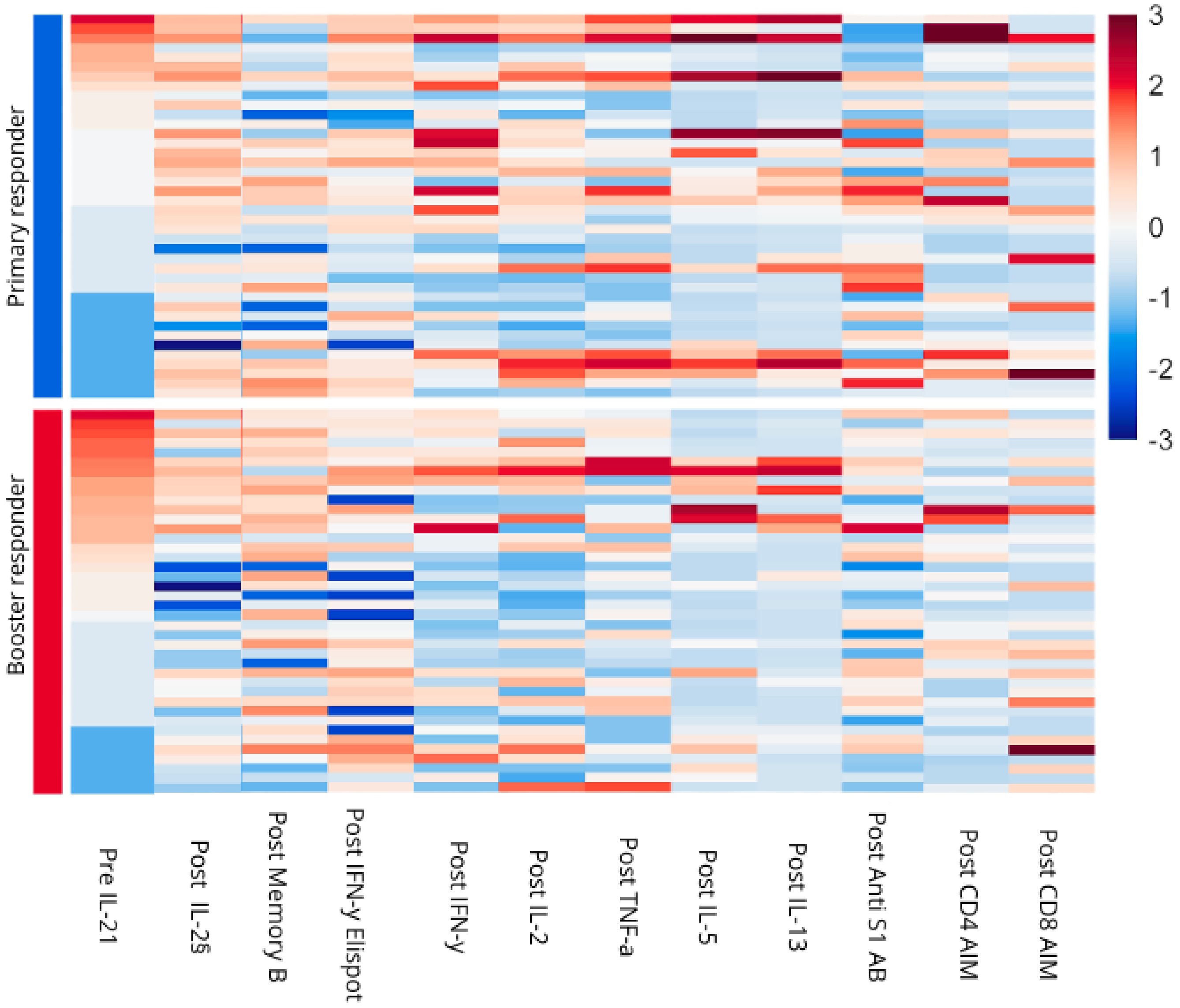

Supplementary Figure 4. Quantitative heatmap of SARS-CoV-2–specific immune responses in kidney transplant recipients. Each row represents an individual KTR, and each column represents a measured immune parameter. Columns are ordered by immune marker, with IL-21 baseline responses shown first, followed by post-vaccination parameters. Rows are grouped by response category, primary responders (blue) and booster responders (red), and within each group sorted by decreasing IL-21 response. Values were log10(x+1)-transformed and scaled per variable (z-score) across all participants. Colors indicate relative expression levels, from low (blue) to high (red).

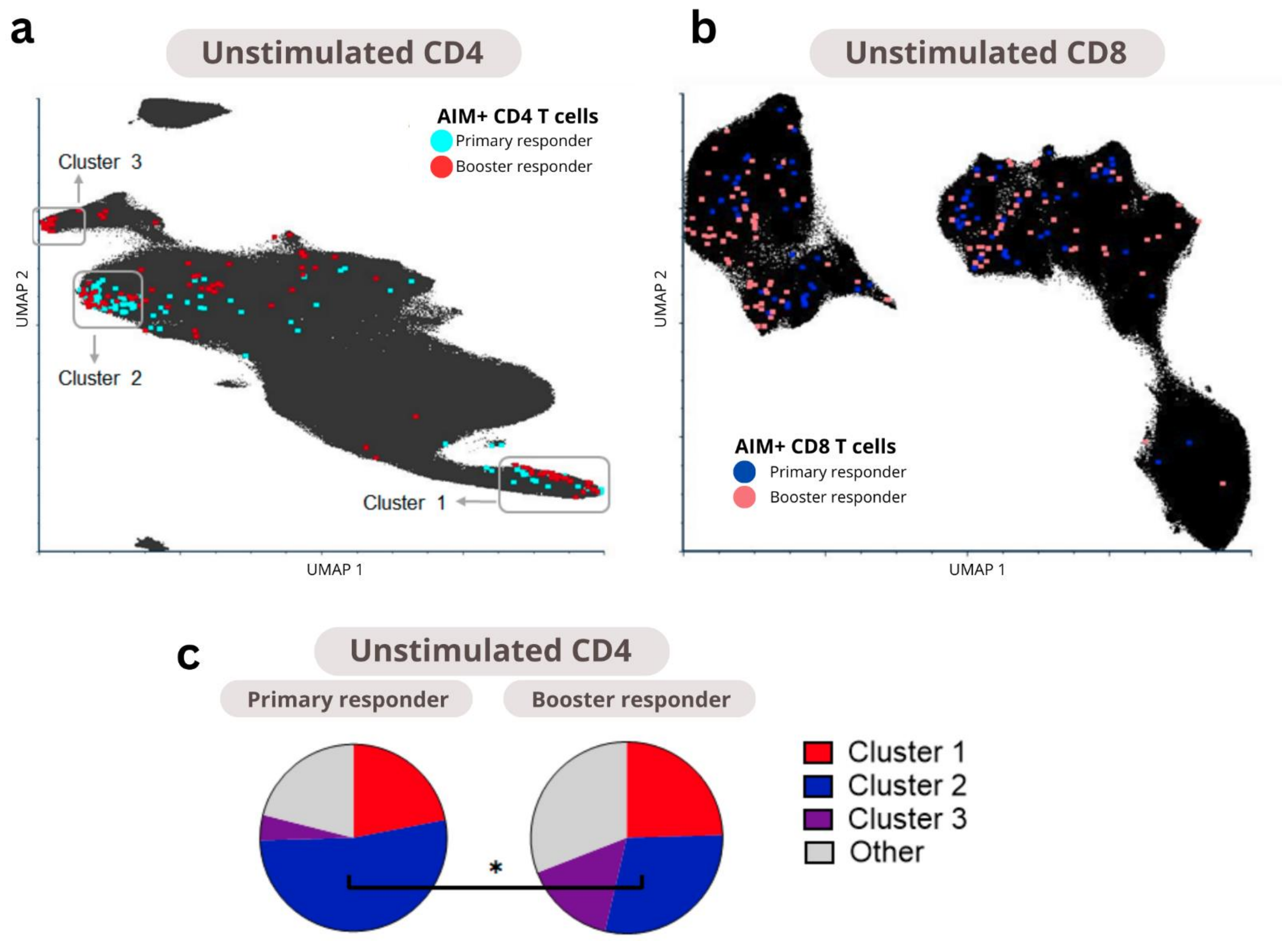

Supplementary Figure 5. AIM+ CD4 and CD8 cells in unstimulated samples of primary and booster responders. **(a)** CD4 UMAP of unstimulated samples and **(b)** CD8 UMAP of unstimulated samples, with the AIM+ cells indicated by the colored dots. The CD4 clusters are also indicated in the unstimulated UMAP. **(c)** Comparison of the proportion of the CD4 AIM+ cells in each cluster in the unstimulated samples, compared between primary and booster responders. Statistical testing by Mann Whitney U. Statistical significance is indicated by asterisks: * $p < 0.05$, ** $p < 0.01$, *** $p < 0.001$, **** $p < 0.0001$.

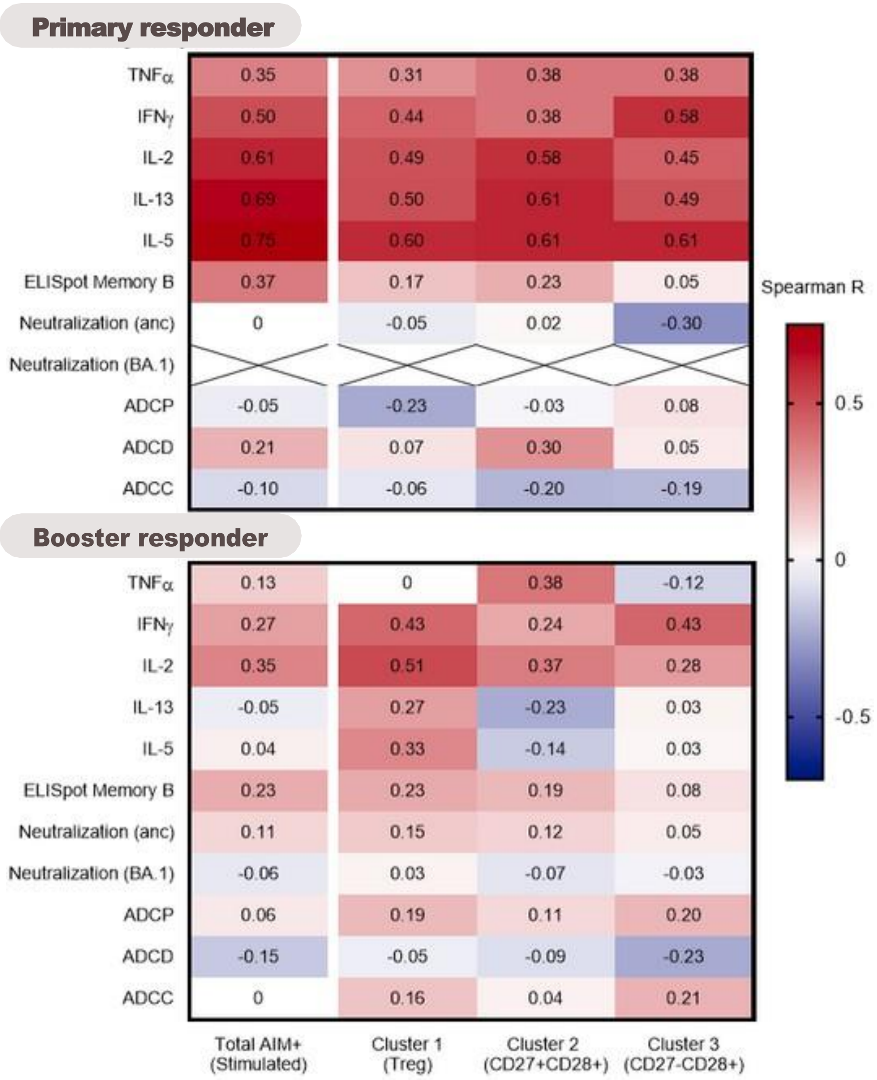

Supplementary Figure 6. AIM+ CD4 cluster associations with B and T cell functional responses in primary and booster responders. The individual frequency of AIM+ cells and cells in the three clusters were correlated with all the additional immunogenicity outcomes in the current study: cytokine levels in the supernatant of the PBMC culture, spot-forming cell counts in the memory B cell ELISpot, ancestral (anc) and BA.1 variant neutralization in the plaque reduction neutralization test, antibody-mediated phagocytosis (ADCP), antibody-dependent complement deposition (ADCD) and antibody-dependent cell- mediated cytotoxicity (ADCC). Correlations by Spearman R.

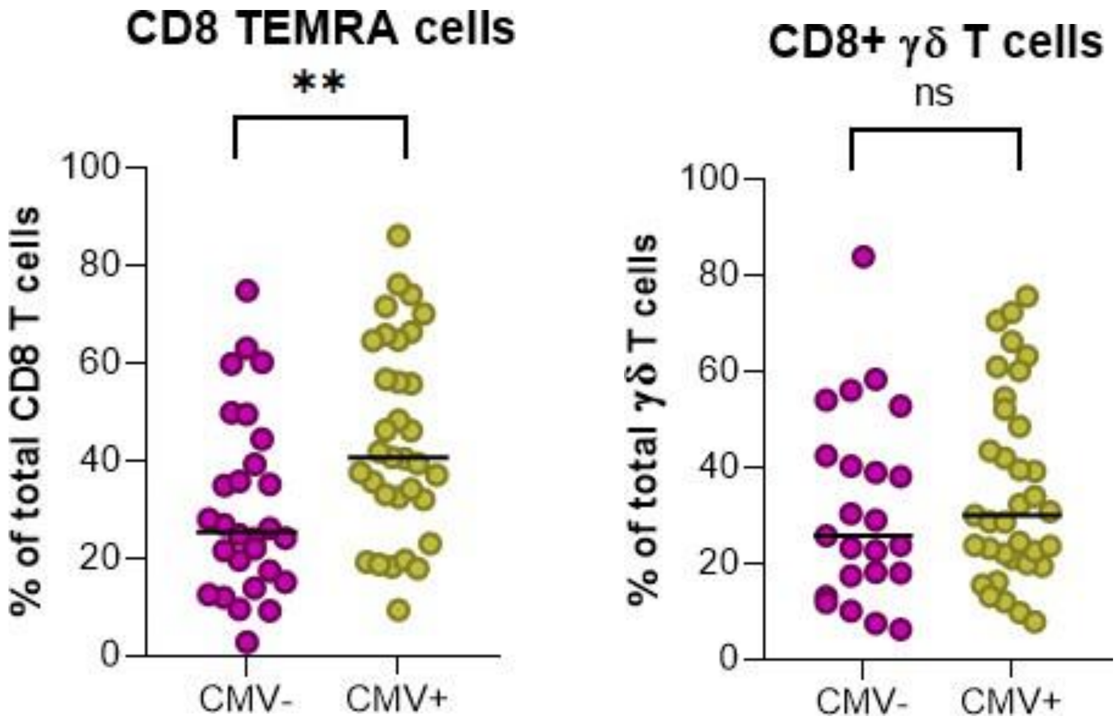

Supplementary Figure 7. Effect of CMV serostatus on the frequency of CD8 TEMRA cells and CD8-expressing γδ T cells. Unstimulated samples of both groups were compared between CMV seronegative (n=23) and CMV seropositive patients (n=35). CMV serostatus was unknown for eight participants, which are therefore not shown. For CD8 TEMRAs, five samples were excluded that displayed an aberrant CD45RA expression that is likely associated with C77G polymorphism. Medians and results of statistical testing by Mann Whitney U are indicated in the graphs. Statistical significance is indicated by asterisks: * $p < 0.05$, ** $p < 0.01$, *** $p < 0.001$, **** $p < 0.0001$.

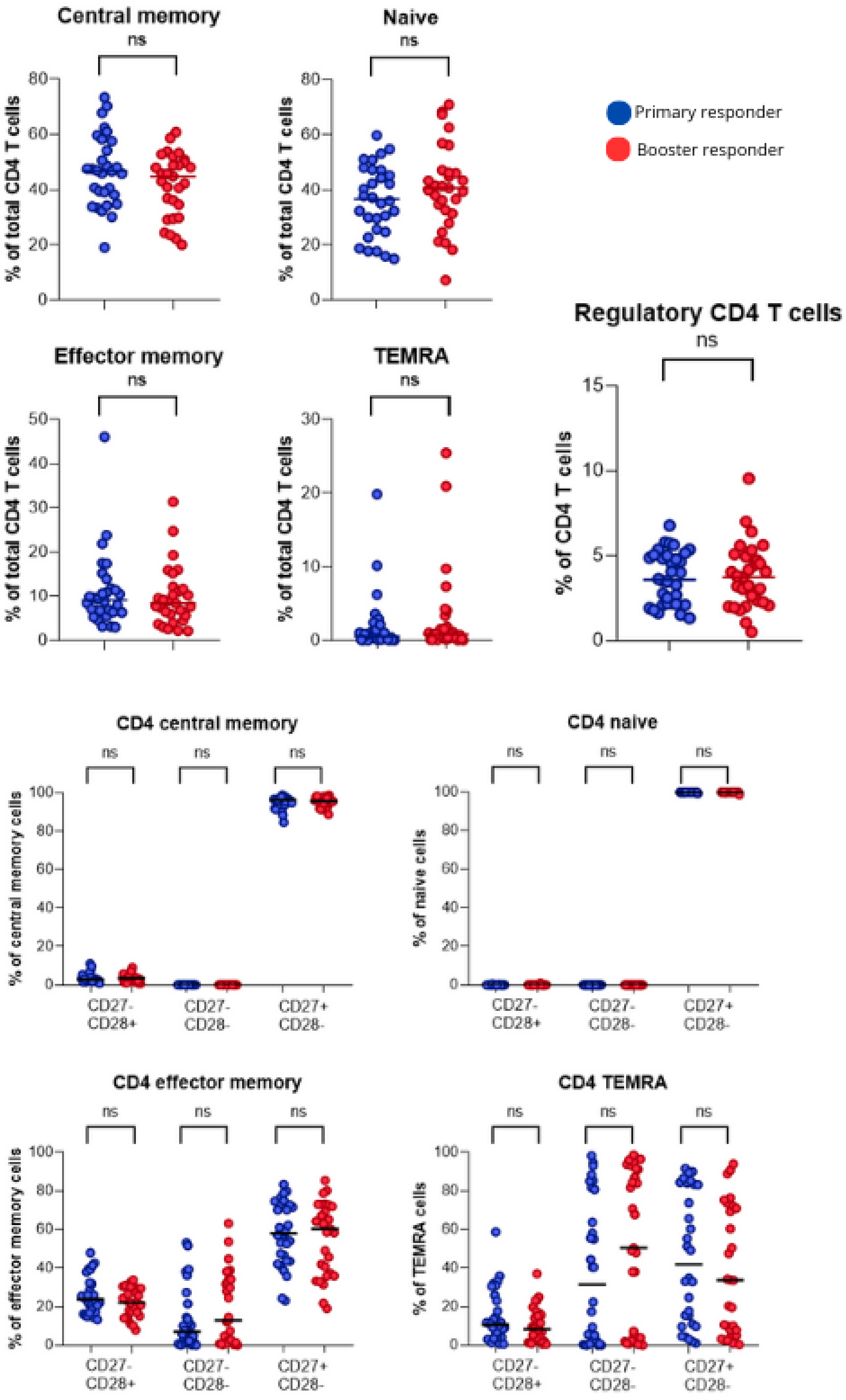

Supplementary Figure 8. Phenotypical differences in CD4 T cell populations between primary and booster responders. Unstimulated samples were compared between primary responders and booster responders. For analyses dependent on CD45RA as gating marker, six samples were excluded that displayed an aberrant CD45RA expression that is likely associated with C77G polymorphism. Medians and results of statistical testing by Mann Whitney U are indicated in the graphs. Statistical significance is indicated by asterisks: * $p < 0.05$, ** $p < 0.01$, *** $p < 0.001$, **** $p < 0.0001$.
